# Behavior of 1-Deoxy-, 3-Deoxy- and *N*-Methyl-Ceramides in Skin Barrier Lipid Models

**DOI:** 10.1038/s41598-020-60754-4

**Published:** 2020-03-02

**Authors:** Andrej Kováčik, Petra Pullmannová, Ludmila Pavlíková, Jaroslav Maixner, Kateřina Vávrová

**Affiliations:** 1 Skin Barrier Research Group, Faculty of Pharmacy in Hradec Králové, Charles University, Akademika Heyrovského 1203, 50005 Hradec Králové, Czech Republic; 2 University of Chemistry and Technology in Prague, Faculty of Chemical Technology, Technická 5, 166 28 Prague, Czech Republic

**Keywords:** Membrane biophysics, Sphingolipids

## Abstract

Ceramides (Cer) are essential components of the skin permeability barrier. To probe the role of Cer polar head groups involved in the interfacial hydrogen bonding, the *N*-lignoceroyl sphingosine polar head was modified by removing the hydroxyls in C-1 (1-deoxy-Cer) or C-3 positions (3-deoxy-Cer) and by *N*-methylation of amide group (*N*-Me-Cer). Multilamellar skin lipid models were prepared as equimolar mixtures of Cer, lignoceric acid and cholesterol, with 5 wt% cholesteryl sulfate. In the 1-deoxy-Cer-based models, the lipid species were separated into highly ordered domains (as found by X-ray diffraction and infrared spectroscopy) resulting in similar water loss but 4–5-fold higher permeability to model substances compared to control with natural Cer. In contrast, 3-deoxy-Cer did not change lipid chain order but promoted the formation of a well-organized structure with a 10.8 nm repeat period. Yet both lipid models comprising deoxy-Cer had similar permeabilities to all markers. *N-*Methylation of Cer decreased lipid chain order, led to phase separation, and improved cholesterol miscibility in the lipid membranes, resulting in 3-fold increased water loss and 10-fold increased permeability to model compounds compared to control. Thus, the C-1 and C-3 hydroxyls and amide group, which are common to all Cer subclasses, considerably affect lipid miscibility and chain order, formation of periodical nanostructures, and permeability of the skin barrier lipid models.

## Introduction

Mammalian skin protects the body from external threats, such as chemicals and ultraviolet light, and prevents excessive water loss. The major skin permeability barrier is located in the outermost layer of the epidermis, the *stratum corneum* (SC)^1–3^. The SC consists of corneocytes, epidermal cells in a terminal phase of differentiation, and extracellular lipid matrix, which is a multilamellar assembly of ceramides (Cer), free fatty acids and cholesterol (Chol) in an approximately 1:1:1 molar ratio, and minor components, such as cholesteryl sulfate (CholS)^1–3^.

Cer, *i.e*., *N*-acyl sphingosines, are simple sphingolipids that have from two to four hydroxyl groups and monosubstituted amide group, which behave as hydrogen bond donors and acceptors and affect Cer interactions with proteins and other lipids. For example, 1-deoxy-Cer do not mix well with other lipids^4^, removal of C-3 hydroxyl in Cer prevents the formation of water-extended cooperative H-bond network of Cer^5^, and Cer amide group appears to be fundamental in creating the signal-transducing membrane platforms^6^. In the skin barrier, additional hydroxyls in Cer at C-4 (phytosphingosine Cer) or C-6 positions (6-hydroxyCer) modulate the lipid miscibility, lamellar arrangement and permeability of model SC lipid mixtures^7–10^, along with the correct acyl chain length^11,12^ or sphingosine chain length of Cer^13,14^.

Topical supplementation of skin lipids in diseases such as atopic dermatitis or ichthyoses is an established therapeutic approach. Such Cer-dominant lipids are safe, can actually prevent inflammation in atopic dermatitis and have corticoid sparing effect (for a review, see ref. ^15^). However, wider use of this approach is hampered by the facts that most native skin Cer are not commercially available or extremely expensive and their isolation from the skin is not possible in sufficient quantities. Thus, a detailed investigation to determine what molecular features of Cer are essential for their functions in the skin barrier can help design less expensive Cer analogues for correcting the skin barrier abnormalities.

**Figure 1:**
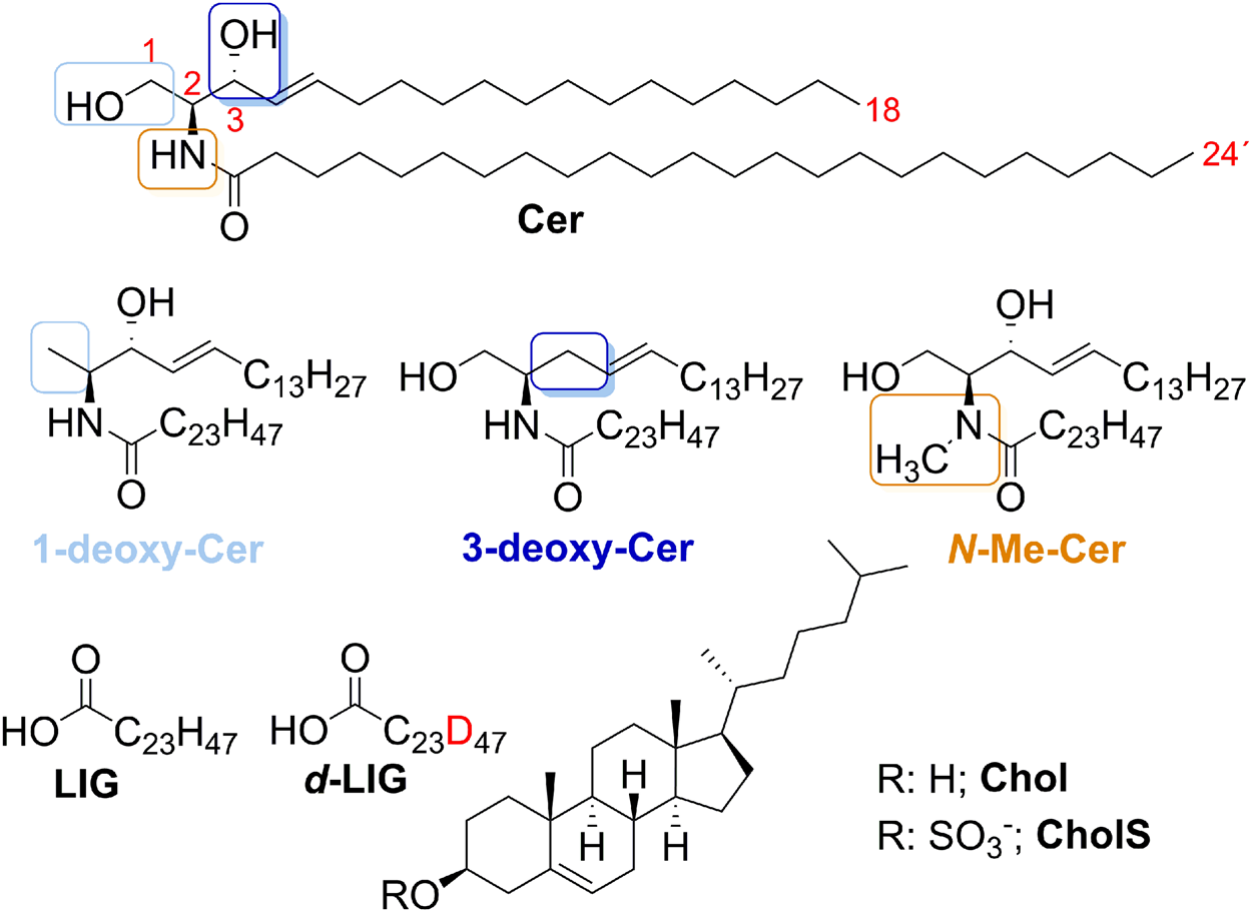
Structure of natural *N*-lignoceroyl sphingosine (Cer NS in Motta nomenclature^16^, referred to as Cer here for brevity), its unnatural analogs, *i.e*., 1-deoxy-Cer (the chemical modification highlighted in light blue), 3-deoxy-Cer (dark blue) and *N*-Me-Cer (orange), and other SC lipids used to construct the lipid barrier models.

Here we focus on the role of the polar head groups that are common to all Cer subclasses, *i.e*., C-1 hydroxyl, C-3 hydroxyl and amide hydrogen, in skin lipid models. *N*-Lignoceroyl sphingosine (CerNS in Motta nomenclature^16^, referred to as Cer here for brevity) was selected as the natural skin Cer for the purpose of this study because it has only the above-mentioned polar head groups (in contrast to the most abundant skin Cer based on phytosphingosine). First, we synthesized unnatural Cer, in which the hydrogen bond donors have been removed: *N*-lignoceroyl 1-deoxy-sphingosine (1-deoxy-Cer), *N*-lignoceroyl 3-deoxy-sphingosine (3-deoxy-Cer) and *N*-lignoceroyl *N*-methyl-sphingosine (*N*-Me-Cer; Fig. 1). Cer (either natural or modified), lignoceric acid (LIG; C24:0, the most abundant SC fatty acid^17^), and Chol in 1:1:1 molar ratio, with 5 weight % CholS were used to construct the lipid models. The nanostructure and thermotropic phase behavior of the lipid mixtures were probed by X-ray diffraction (XRD) and infrared spectroscopy with either unlabeled LIG or deuterated *d*-LIG. The barrier properties of the SC lipid models were studied using four permeability markers: water loss, flux of theophylline (TH), flux of indomethacin (IND), and electrical impedance.

## Results

### Synthesis of Cer analogs and preparation of SC lipid models

The studied Cer analogs were synthesized from the respective sphingoid bases (1-deoxy-sphingosine, or 3-deoxy-sphingosine, or *N*-methyl-sphingosine) and LIG using WSC and HOBt^18^ in the following yields: 94% for 1-deoxy-Cer, 69% for 3-deoxy-Cer and 71% for *N*-Me-Cer (^1^H and ^13^C NMR spectra are shown in Supporting Fig. S1). The removal of C-3 hydroxyl group and *N-*methylation decreased the melting temperatures of 3-deoxy-Cer (81–84 °C) and *N*-Me-Cer (46–48 °C), whereas the melting point of 1-deoxy-Cer (92–94 °C) was comparable to the natural Cer (89–91 °C; Supporting Fig. S2). The retention on silica gel of the prepared Cer was as follows: 1-deoxy-Cer (retention factor = 0.80) > 3-deoxy-Cer (0.75) > Cer (0.44) > *N*-Me-Cer (0.25). The synthesized unnatural Cer analogs along with the parent sphingosine-based Cer (all Cer with C24:0 acyl chains, the most abundant chain length in skin sphingolipids^17^) were used for the preparation of the SC lipid models composed of Cer/LIG/Chol in a 1:1:1 molar ratio, with an addition of 5 wt% CholS^19^. The samples were prepared by spraying a lipid solution on a substrate, annealing at 90 °C for 10 min, and slowly (⁓4 h) cooling to room temperature. Then the samples were maintained at 32 °C for 3 days. Whether this procedure ensures an equilibrium or near-equilibrium state of the samples is unknown. However, previous experiments showed that permeabilities^12^ and XRD patterns (unpublished) did not change over at least 14 days. In addition, kinetic experiments showed that Cer/fatty acid/Chol lipid mixtures either separated into domains within hours or, in case of hydrophobically matched Cer NS24/LIG, remained stable for 200 h^20,21^.

### Lamellar and lateral organization of SC lipid models

**Figure 2:**
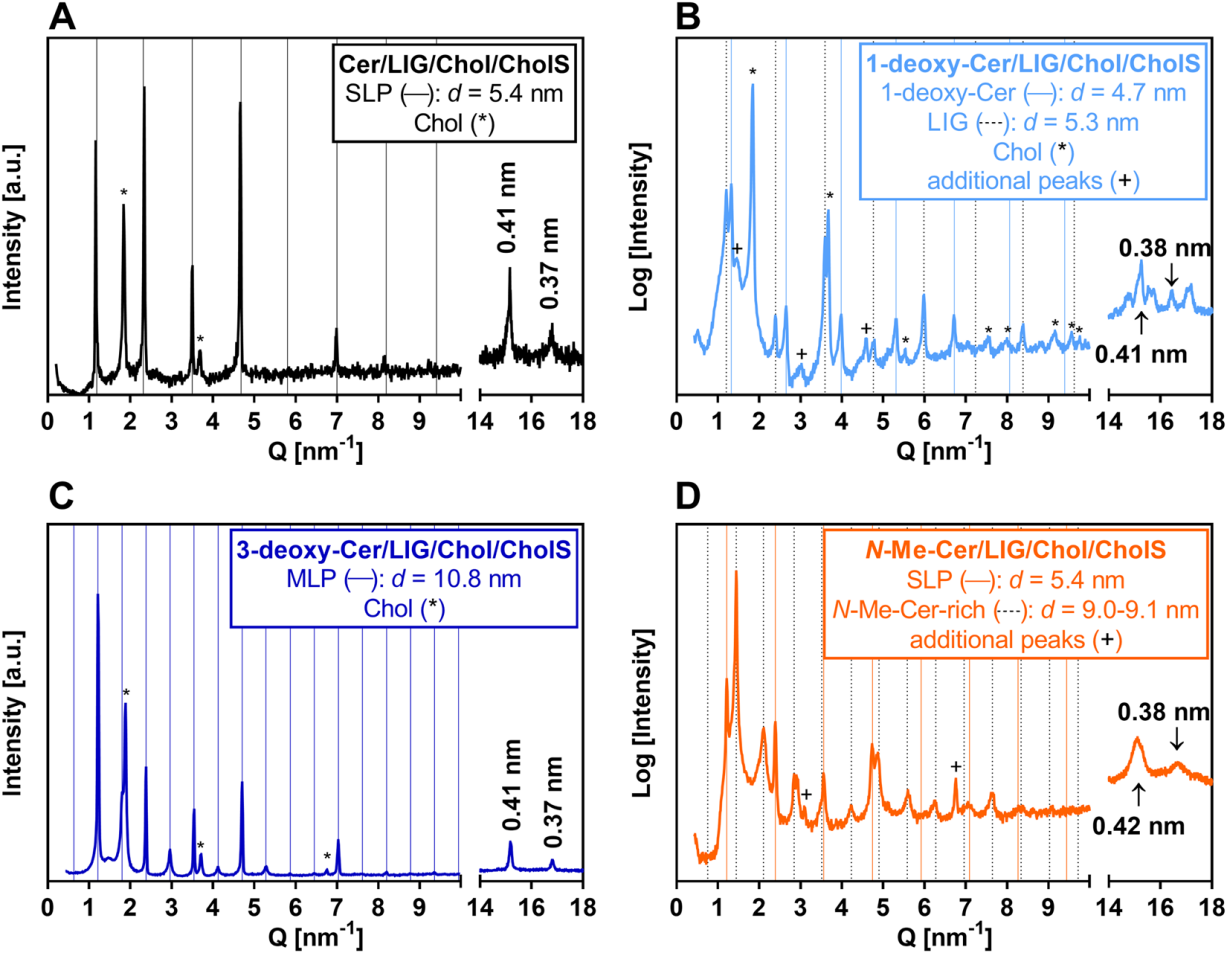
XRD diffractograms of SC lipid models containing either Cer (panel A), or 1-deoxy-Cer (B), or 3-deoxy-Cer (C), or *N*-Me-Cer (D), along with LIG, Chol, and CholS. The intensity is given in arbitrary units (a. u.). Full and dashed grid lines predict the positions of SLP (panel A and D), MLP (panel C), and other structure reflections. Asterisks mark the separated Chol. The determined repeat distances are shown in the respective panels. The peaks at 0.41 nm and 0.37 nm in the wide-angle region (Q = 14–18 nm^−1^) indicate the orthorhombic packing of polymethylene lipid chains.

The effects of the modified Cer on the lamellar phases and lateral packing in the lipid mixtures were investigated using XRD (Fig. 2, and Supporting Tables S2–S4). To aid the interpretation of the detected phases, additional samples were prepared from pure Cer analogs and from the mixtures of Cer analogs/Chol (1:1 mol/mol) using the same preparation protocol (Supporting Fig. S3**)**. The repeat distances, *d*, from two parallel samples did not differ by more than 0.01 nm, unless specified otherwise. The diffractogram of the control sample composed of physiological Cer/LIG/Chol/CholS showed a set of diffraction peaks that gave *d* = 5.40–5.41 nm (Fig. 2A and Supporting Table S1). The *d* of this short lamellar phase (SLP) is consistent with those found in analogous SC lipid models containing LIG^8^ or a mixture of free fatty acids with C16-C24 chain lengths^9,22,23^. In addition, a separated Chol phase with *d*_(001)_ = 3.41 nm was found. Such separated Chol phase has also been found in the SC models^12,24^ and in the human SC^25^.

The removal of C-1 hydroxyl of Cer resulted in extensive phase separation of lipid species. Separated Chol and phases giving *d* = 4.68–4.71 nm and *d* = 5.25–5.26 nm have been found in the XRD pattern of the 1-deoxy-Cer–based model (Fig. 2B and Supporting Table S2). Separated crystalline Chol provided a set of reflections, which were attributed to Chol monohydrate triclinic lattice (space group P1^26^). The reflections of the 5.25 nm phase were attributed to separated crystalline LIG by comparing their positions with the crystallographic data of long-chain fatty acids^27^. The phase with *d* ~4.7 nm corresponded most likely to 1-deoxy-Cer as pure 1-deoxy-Cer and 1-deoxy-Cer/Chol (1:1 mol/mol) mixture formed similar phases with *d* = 4.72 nm and 4.73 nm, respectively (Supporting Fig. S3). Notably, this ~4.7 nm phase is shorter than the approximate length of fully extended 1-deoxy-Cer molecule (~5.3 nm). Such structure can correspond to regularly arranged 1-deoxy-Cer molecules in V-shape conformation, similar to that proposed previously for *N*-(α-hydroxyoctadecanoyl)-phytosphingosine with unnatural L-configuration in α-position^28^.

The 3-deoxy-Cer-based lipid models showed, beside separated Chol, a set of reflections giving *d* = 10.80 nm (Fig. 2C and Supporting Table S3). The XRD pattern of this medium lamellar phase (MLP) contained more intense even orders (2, 4, … up to 18) and less intense odd reflections (3, 5, … up to 11) similar to MLP previously detected in the lipid mixtures with natural Cer^22^. The 1^st^ order reflection was not resolved due to the technical reasons thoroughly discussed elsewhere^22^. The presence of LIG was essential for the formation of MLP as the mixture of 3-deoxy-Cer/Chol contained separated Chol and a crystalline structure providing the repeat distance of 5.54–5.55 nm and 3.72–3.73 nm (Supporting Fig. S3).

The *N*-Me-Cer/LIG/Chol/CholS sample provided sets of reflections with *d* = 5.35 nm (SLP) and *d* = 8.98–9.12 nm (Fig. 2D and Supporting Table S4). Interestingly, the reflections typical for separated Chol disappeared. Pure *N*-Me-Cer formed a lamellar phase with *d* = 5.45–5.47 nm, whereas *N*-Me-Cer/Chol mixture had two phases with *d* = 5.43 nm and 9.20 nm along with separated Chol (Supporting Fig. S3).

In addition to the phases described above, the XRD patterns contained weak additional peaks, which could not be unambiguously assigned to any structure(s). All these unassigned peaks are listed in bold in the Supporting Tables S2–S4. For example, the 1-deoxy-Cer-based lipid film contained additional weak peaks at Q ~ 1.5, 3.0 and 4.6 nm^−1^. The assignment of any reflection requires to consider it in the context of other reflections present in the pattern and reflections observed in previous measurements, and to compare it with crystallographic data, if such data exist. Here, we feel it would be speculative to assign a solitary reflection or an incomplete set of reflections without an appropriate evidence.

The wide-angle regions of the diffractograms at the scattering vector Q = 14–18 nm^−1^ contain information about the polymethylene chain packing. Two peaks at the positions corresponding to distances between the scattering planes of 0.41 and 0.37 nm were found (Fig. 2), that indicate an orthorhombic chain packing (or a co-existence of hexagonal and orthorhombic chain packing)^29^. In human SC, orthorhombically and hexagonally-packed lipids coexist as well^30^. However, these reflections were poorly resolved in the pattern of the *N*-Me-Cer-based models and they coexisted with other reflections in the pattern of the 1-deoxy-Cer-based mixtures.

### Lipid chain order at 32 °C

**Figure 3:**
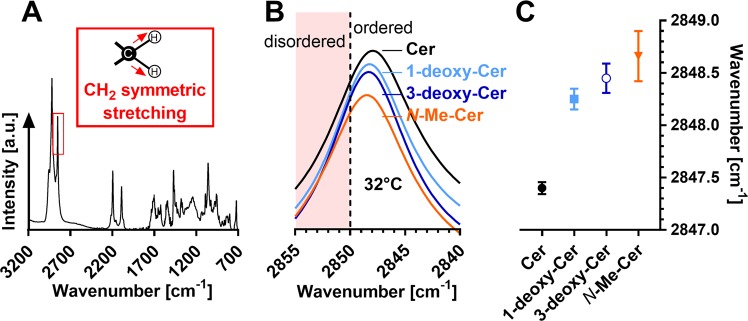
Lipid chain order in the SC lipid models containing Cer (black), 1-deoxy-Cer (light blue), 3-deoxy-Cer (dark blue), *N*-Me-Cer (orange), along with LIG, Chol, and CholS. Panels A and B show an example infrared spectrum and enlarged methylene symmetric stretching vibrations at 32 °C, respectively. Panel C shows the means ± SEM of the wavenumbers of the methylene symmetric vibrations; *n* = 2–3.

Next, the lipid chain order, packing, mixing and phase transitions in the SC lipid models were investigated using infrared spectroscopy^7,31,32^. Lipid conformation order and phase transitions can be deduced from the methylene symmetric stretching vibrations – a shift to higher wavenumbers indicates lipid disordering^33^. In healthy human skin, lipid chains are highly ordered as indicated by a mean wavenumber of 2848.8 cm^−1^ ^34^. The lipid chains in the studied samples were well ordered with high proportions of all-*trans* conformers (wavenumbers below 2850 cm^−1^)^35^ at skin temperature (32 °C) (Fig. 3A,B). 1-Deoxy-Cer, 3-deoxy-Cer, and *N*-Me-Cer decreased the overall chain order compared to samples with natural Cer, as indicated by 0.9–1.3 cm^−1^ shifts in the methylene symmetric stretching to higher wavenumbers (Fig. 3C). Such shifts are rather large in the context of human skin lipids; for example, atopic dermatitis patients have by 0.4 cm^−1^ higher methylene symmetric stretching wavenumbers compared to healthy individuals^34^. However, all these methylene stretching wavenumbers still correspond to solid (or gel) lipid chains as liquid crystalline lipids have characteristic wavenumbers around 2852 cm^−1^ ^36^.

**Figure 4:**
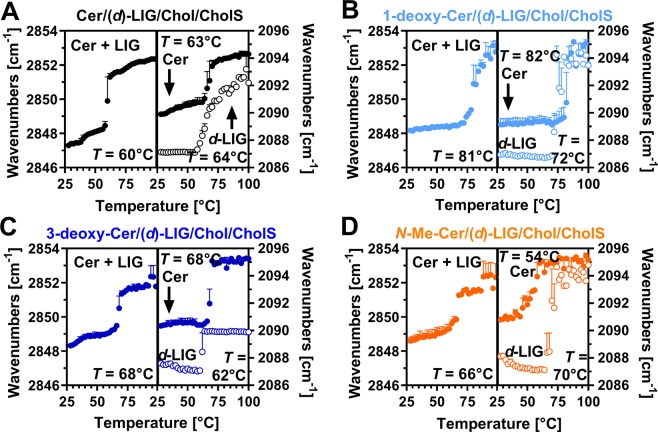
Lipid chain order and phase transitions in the SC lipid models containing the studied Cer (black; A), 1-deoxy-Cer (light blue; B), 3-deoxy-Cer (dark blue; C), or *N*-Me-Cer (orange; D), along with (*d*)-LIG, Chol, and CholS. First graphs in each panel show the unlabeled lipid mixtures, *i.e*., the thermal evolution of infrared symmetric methylene stretching vibrations. Second graphs in each panel show the lipid mixtures with *d*-LIG, *i.e*., the temperature dependence of the symmetric CH_2_ (mainly from Cer, filled circles) and CD_2_ stretching vibrations (from *d*-LIG, open circles). The temperatures of the main phase transitions (estimated from the first derivatives of the curves) are indicated. Data are shown as means ± SEM; *n* = 2–3.

Replacement of unlabeled LIG by deuterated *d*-LIG enables to compare the behavior of the unlabeled methylene chains (mainly from Cer) and deuterated methylene chains (*d*-LIG; second graphs in Fig. 4). The methylene symmetric stretching wavenumbers suggested that in the control sample at 32 °C, both Cer (2849 cm^−1^) and *d*-LIG chains (2087 cm^−1^) were well ordered. The increased CH_2_ stretching wavenumber in this sample compared to that with unlabeled LIG was likely caused by isotopic dilution that disturbs the interchain coupling and results in such shift to higher wavenumber (while C-D bonds are less affected)^37^. Removal of C-3 hydroxyl did not change the wavenumbers of either 3-deoxy-Cer or *d*-LIG relative to the control. In the *N*-Me-Cer sample, the methylene symmetric stretching wavenumbers of both *N*-Me-Cer (2850 cm^−1^) and *d*-LIG chains (2089 cm^−1^) were by 1–2 cm^−1^ higher than those in the control Cer sample. In contrast, in 1-deoxy-Cer-based samples, the CH_2_ stretching wavenumbers were similar with LIG and *d-*LIG (2848 cm^−1^) indicating well ordered 1-deoxy-Cer chains and their limited mixing with (*d-*)LIG.

### Phase transitions and lipid mixing

The thermal evolution of the methylene symmetric stretching vibrations can be used to determine phase transitions (as the lipids disorder upon heating, their methylene stretching modes shift to higher wavenumbers). We estimated the inflection points of the transition from the first derivatives of the curves. The lipids in Cer/LIG/Chol/CholS-containing samples first underwent a 0.2 cm^−1^ shift of the methylene symmetric stretching to higher wavenumbers at 37 °C (Fig. 4). This pre-transition was followed by an order-to-disorder transition at 60 °C, as indicated by a sharp shift of the wavenumber to over 2850 cm^−1^, which is in good agreement with previous studies^8,12,38^. Replacement of the unlabeled LIG by deuterated *d*-LIG showed that both Cer (CH_2_) and *d*-LIG (CD_2_) became disordered at similar temperatures (63 °C for Cer and 64 °C for *d*-LIG; Fig. 4A).

The lipid chains in the 1-deoxy-Cer/LIG/Chol/CholS sample (Fig. 4B) did not show any disordering up to 75 °C. Subsequently, a relatively sharp phase transition occurred at 81 °C, which is by 21 °C higher than in the control sample with natural Cer. 1-Deoxy-Cer chains and *d-*LIG became disordered at 83 °C and 72 °C, respectively (Fig. 4B), indicating their phase separation. In the 3-deoxy-Cer/LIG/Chol/CholS sample (Fig. 4C), the methylene symmetric stretching wavenumbers first increased by 0.5 cm^−1^ at 42 °C. The main order-to-disorder phase transition occurred at 68 °C. In the analogous lipid mixture with *d*-LIG, phase transition temperatures of 3-deoxyCer (68 °C) and *d*-LIG (62 °C) were rather close to those found in the control sample. In the model sample composed of *N*-Me-Cer/LIG/Chol/CholS, the methylene symmetric stretching wavenumbers increased slowly with increasing temperature but without any apparent pre-transition (Fig. 4D). The main phase transition was rather broad (over more than 15 °C; we estimated its inflection point at approximately 66 °C), which indicates co-existence of two or more phases, in agreement with XRD results. In the model mixture with *d*-LIG, the phase transitions of the CH_2_ chains (54 °C) and CD_2_ chains (70 °C) were clearly separated. However, the slight decrease of CD_2_ wavenumber before the main transition suggests some rearrangement of the *d-*LIG organization before its actual melting.

### Lateral chain packing and lipid mixing

**Figure 5:**
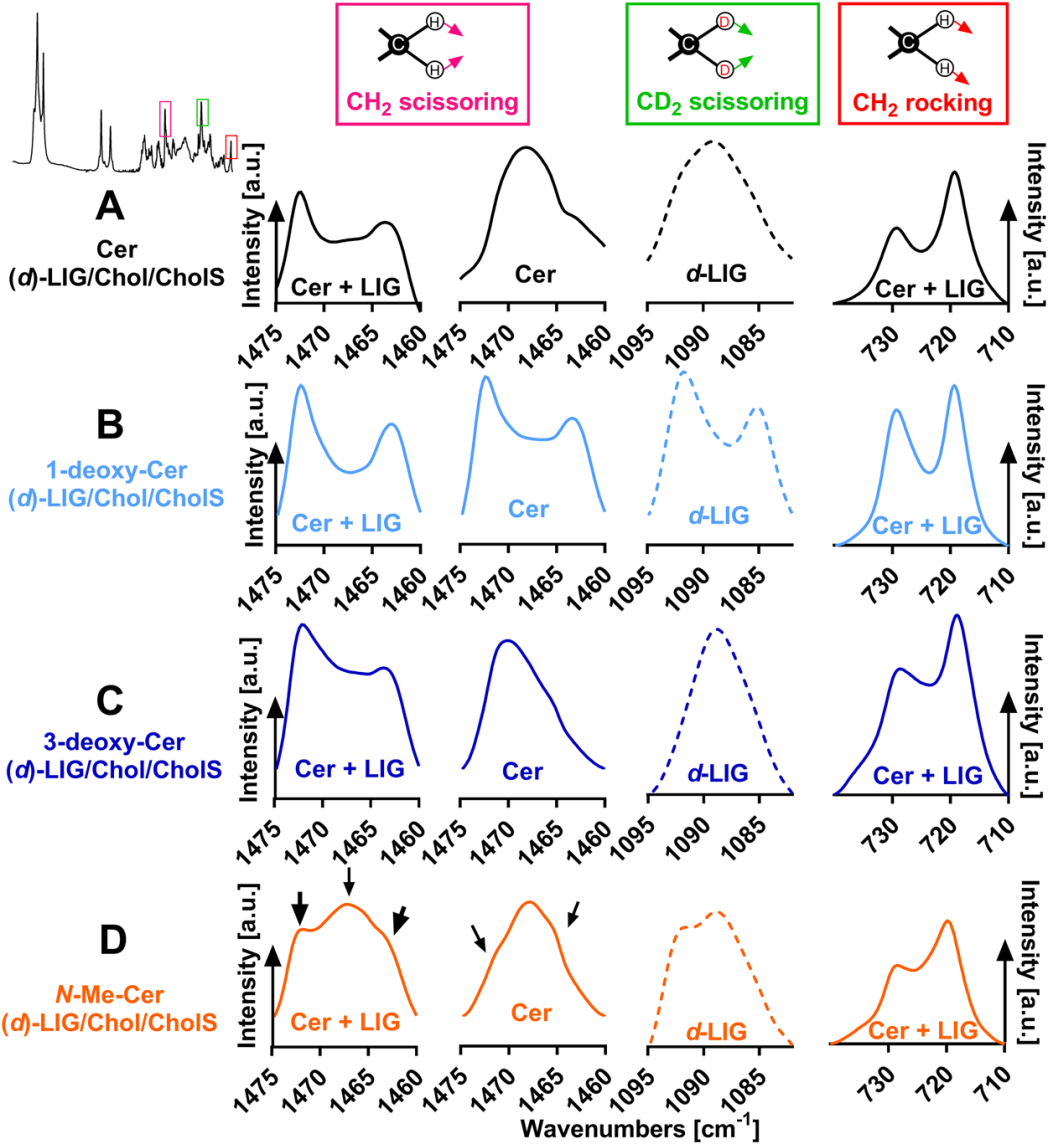
Lateral lipid packing in the SC lipid model containing either Cer (panel A), or 1-deoxy-Cer (panel B), or 3-deoxy-Cer (panel C), or *N*-Me-Cer (panel D), along with (*d*)-LIG, Chol, and CholS. First graphs in each panel show the representative infrared spectra of the methylene scissoring vibrations of the unlabeled lipid mixtures at 32 °C. Second and third graphs in each panel show the CH_2_ scissoring vibration (mainly from Cer) and CD_2_ scissoring vibrations (*d*-LIG; dashed line), respectively, of the lipid mixtures with *d*-LIG at 32 °C. The fourth graphs in each panel show the representative infrared spectra of the methylene rocking vibrations of the unlabeled mixtures at 32 °C. A presence of a CH_2_/CD_2_ doublet in the spectrum is indicative of an orthorhombic packing. Intensity is given in arbitrary units (a. u.).

The infrared methylene rocking and scissoring bands are sensitive to the lateral packing of the lipid chains^39^. The splitting of the bands into doublets is indicative of an orthorhombic chain packing because the vibrations in this tight lateral packing are coupled^35,40^. Fig. 5 (first graphs in each panel) shows the methylene scissoring vibrations at 32 °C; the thermal evolution of the most prominent bands in this region is given in Supporting Fig. S4. Note that the relative extent of orthorhombic packing may be also affected by the relative proportions of lipids undergoing the orthorhombic-hexagonal transition. However, this comparison at 32 °C gives an estimate of the lateral packing at the temperature, at which the permeability studies were performed.

All the studied lipid models had orthorhombic doublets at approximately 1462 and 1472 cm^−1^, which is consistent with XRD data. However, in the *N*-Me-Cer lipid mixture, a central band at approximately 1468 cm^−1^ attributed to hexagonal packing^35^ dominated the scissoring region. Such coexistence of orthorhombic and hexagonal phases has also been found in human SC^30^. The methylene rocking bands (doublets at approximately at 719 and 729 cm^−1^) confirmed the presence of the orthorhombic lipid chain packing (fourth graphs in Fig. 5A–D). The ratios of the 729–719 cm^−1^ peak intensities that roughly estimate relative orthorhombic packing ratios (Supporting Fig. S4) were as follows: 1-deoxy-Cer > Cer = 3-deoxy-Cer > *N*-Me-Cer.

The orthorhombic packing disappeared with increasing temperature, either before the main transition (at 37 °C in the Cer sample) or at the onset of the main transition: at 75 °C in sample with 1-deoxy-Cer, at 65 °C with 3-deoxy-Cer, and at 51 °C with *N*-Me-Cer (Supporting Fig. S4, first graphs in each panel). Notably, the scissoring doublets in the 3-deoxy-Cer and 1-deoxy-Cer samples first became narrower at 42 °C and 52 °C, respectively, before dissolving into a singlet. This behavior suggests a decreased size of the orthorhombic domains and is consistent with the pre-transition at 42 °C detected in methylene symmetric stretching vibration.

The lipid chain deuteration shifts the methylene scissoring wavenumbers to approximately 1090 cm^−1^. Thus, the behavior of the CH_2_ and CD_2_ chains could be individually examined. In the control model lipid sample, *i.e*., Cer/*d*-LIG/Chol/CholS, CH_2_ and CD_2_ singlets were found at approximately 1468 and 1089 cm^−1^, respectively (second and third graphs in Fig. 5A). Thus, the scissoring doublet observed in the unlabeled sample changed into singlets upon LIG deuteration, which indicates a good mixing of *d*-LIG and Cer. This is because the vibrational coupling does not occur between different isotopes^35^. Similar scenario was observed in the 3-deoxy-Cer sample: the prevalent singlets at approximately 1090 and 1471 cm^−1^ suggested mostly good mixing of 3-deoxy-Cer with LIG. Notably, the CH_2_ singlet was rather distorted indicating that a small proportion of lipid chains was separated.

In contrast, the absence of C-1 hydroxyl in 1-deoxy-Cer resulted in a separation of rather large domains of 1-deoxy-Cer and *d*-LIG, as indicated by well-defined CH_2_ and CD_2_ doublets. The 1-deoxy-Cer doublet collapsed at 33 °C, whereas the *d-*LIG doublet persisted to 69 °C, the onset of its main transition (Supporting Fig. S4B). In the model mixture with *N*-Me-Cer, small orthorhombic *d*-LIG domains were deduced from a doublet at approximately 1093 and 1089 cm^−1^). This orthorhombic packing collapsed at 67 °C, with the onset of the *d*-LIG disordering (Supporting Fig. S4D). The carboxyl vibrations in the control Cer and 3-deoxy-Cer mixtures were located at 1718–1719 cm^−1^ at 32 °C, whereas they were shifted to lower wavenumbers (1703–1704 cm^−1^) in the 1-deoxy-Cer and *N*-Me-Cer samples indicating their stronger hydrogen bonding, which may be connected with the suggested LIG separation in the latter two samples (Supporting Fig. S5).

### Transmembrane water loss

**Figure 6:**
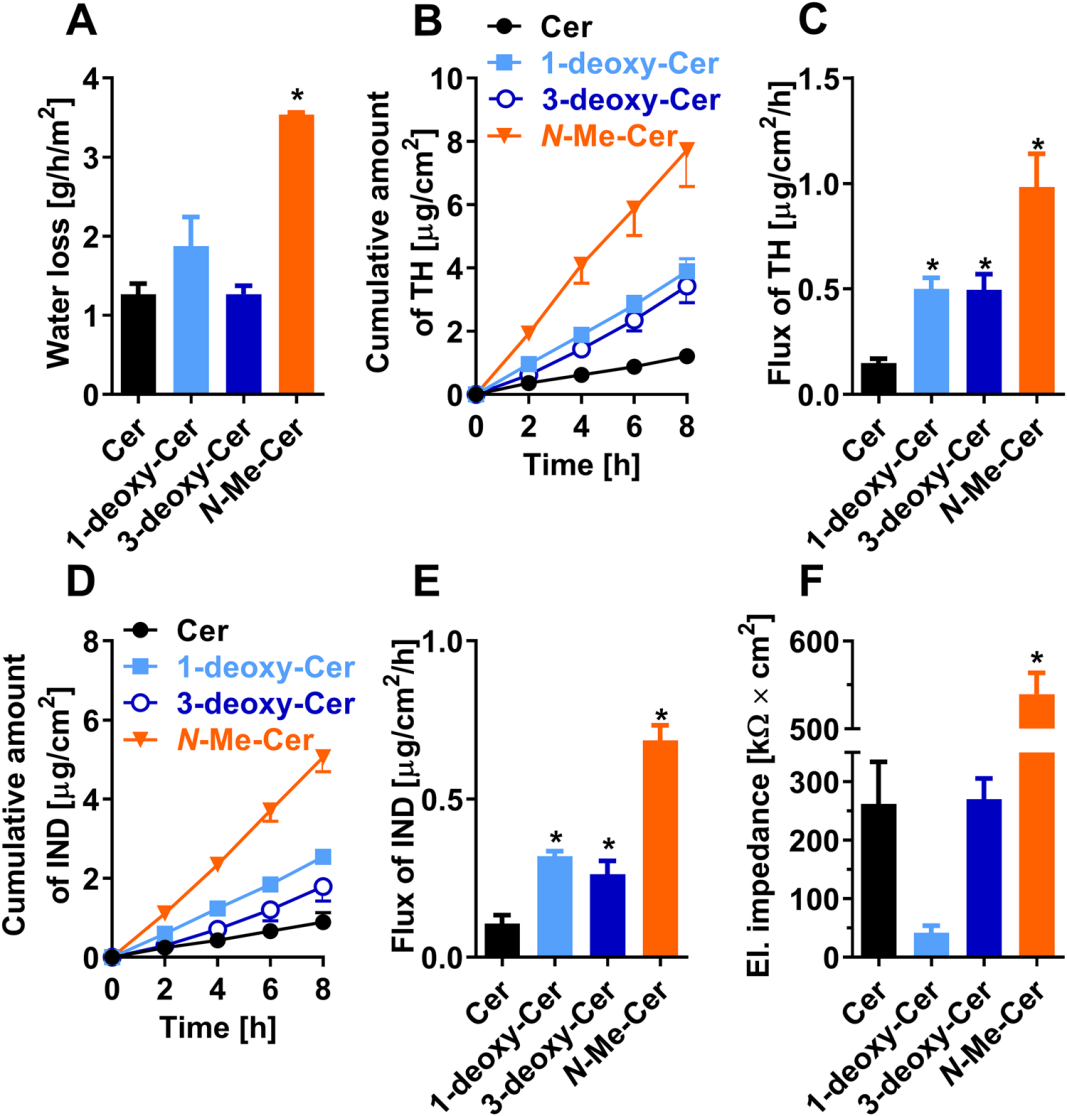
The permeabilities of the studied SC lipid models composed either of Cer (black), or 1-deoxy-Cer (blue), or 3-deoxy-Cer (dark blue), or *N*-Me-Cer (orange), along with LIG, Chol, and CholS. (**A**) Water loss; (**B**) permeation profile for TH; (**C**) the flux of TH; (**D**) permeation profile for IND; (**E**) the flux of IND; (**F**) electrical impedance. Data are presented as the means ± standard error of the mean (SEM), *n* = 5–16. The asterisks show statistically significant differences against the Cer-containing sample (*p* <0.05).

Next, the effects of the studied structural changes in Cer molecule on the permeabilities of the SC lipid models were probed using four markers. First, the water loss through the lipid films was investigated (Fig. 6A)^19^. This method is mostly used to evaluate the skin barrier condition *in vivo*^41,42^, but is also useful in *in vitro* studies on skin^43–45^ and model systems^43,46^. The water loss through the control sample, *i.e*., Cer/LIG/Chol/CholS, was 1.29 ± 0.11 g/h/m^2^, which is consistent with the value published previously (1.40 ± 0.10 g/h/m^2^)^9^. A direct comparison of the water loss through the membrane and skin is not possible because of the differences in the instrument used, lipid amount, absence/presence of corneocytes, sweat glands, etc, but these values are mostly within the same order of magnitude^34,47^. Model SC mixture based on 1-deoxy-Cer had apparently (by 46%, not significant at p < 0.05) higher water loss than control. For example, in atopic dermatitis, water loss is mostly 2–3-fold higher compared to healthy individuals^34,48,49^. The deoxygenation of Cer in position 3 did not change the permeability of our model systems to water. In contrast, methylation of the amide nitrogen significantly (almost 3-fold) increased the water loss through the samples composed of *N*-Me-Cer/LIG/Chol/CholS (Fig. 6A).

### Permeability to TH and IND

Next, we investigated the permeabilities of the lipid models to two model permeants, TH and IND. Note that these substances were selected solely because of their different physicochemical properties without any potential clinical implications. The permeation profiles and steady-state fluxes of the model permeants are shown in Fig. 6B–E. TH is a small molecule (M_w_ = 180.2 g/mol) with balanced lipophilicity (log*P* = 0), which is likely to cross lipid membranes via free-volume diffusion^50^. The steady-state flux of TH for the control model sample, *i.e*., Cer/LIG/Chol/CholS, was 0.11 ± 0.02 µg/h/cm^2^. This is in good agreement with published data^9^. The permeability coefficient for TH through these solid lipid sample (1.22 × 10^−9^ cm.s^−1^; calculated according to Mitragotri *et al*.^51^ from the TH flux and its concentration in the donor sample^52^) is approximately 5 orders of magnitude lower compared to its permeability through a fluid bilayer (2.9 × 10^−4^ cm.s^−1^ has been reported for egg yolk phosphatidylcholine-based membrane^53^). The replacement of Cer by its analogs 1-deoxy-Cer, 3-deoxyCer and *N*-Me-Cer significantly increased TH flux to 0.50 ± 0.02 µg/h/cm^2^, 0.49 ± 0.05 µg/h/cm^2^, and 0.98 ± 0.16 µg/h/cm^2^, respectively (Fig. 6B–C). IND is a larger (M_w_ = 357.8 g/mol) and more lipophilic molecule (log*P* = 4.3) than TH, and prefers lateral diffusion along lipid layers^50^. The IND flux values through the samples based on Cer, 1-deoxy-Cer, 3-deoxy-Cer and *N*-Me-Cer were 0.06 ± 0.01 µg/h/cm_2_ (comparable with literature^9^), 0.32 ± 0.01 µg/h/cm^2^, 0.26 ± 0.02 µg/h/cm_2_ and 0.69 ± 0.05 µg/h/cm^2^, respectively (Fig. 6D9E). To put these numbers in context, skin permeability to various substances is 2–3.5-fold higher in atopic dermatitis patients compared to healthy volunteers (reviewed in ref. ^54^).

In addition, electrical impedance of the lipid films suggested that they did not have any macroscopic defects (Fig. 6F). The control and 3-deoxy-Cer membranes had electrical impedance of approximately 260–270 kΩ × cm^2^. The impedance of 1-deoxy-Cer membranes was approximately 40 kΩ × cm^2^ (which is higher than usual impedance values of *in vitro* human skin)^55^. This 6-fold decreased opposition to electrical current of 1-deoxy-Cer membranes compared to control can be explained by phase separation in that membrane. Interestingly, *N*-Me-Cer membranes had electrical impedance >500 kΩ × cm^2^. Thus, we can exclude that macroscopic defects are responsible for the high permeability of these samples to water, TH and IND. This high opposition to electrical current may be related to the improved mixing of Chol in the *N*-Me-Cer membranes compared to controls. Nevertheless, we cannot distinguish between the transport properties of a homogeneous structure and local defects/grain boundaries between different crystalline/ordered domains. The samples create different number of phases with periodical arrangement, and can have different crystallinity and mosaicity. Thus, we monitor the permeability of the samples as a sum of all contributing factors.

## Discussion

### 1-deoxy-Cer

Cer lacking the primary hydroxyl at C1 occur at certain pathological conditions (*e.g*., hereditary sensory and autonomic neuropathy type I disease, diabetes, non-alcoholic steatohepatitis^56^), when the common substrate of serine palmitoyl transferase, L-serine, is replaced by L-alanine^56^. Such 1-deoxy-Cer cannot be metabolized to complex sphingolipids because the C-1 hydroxyl is essential for Cer binding to glucose in glucosylCer or to phosphocholine residue in sphingomyelin.

In the skin lipid models studied here, the C-1 hydroxyl in Cer with C24 acyl chain was essential for proper Cer miscibility with other SC lipids. As 1-deoxy-Cer and LIG have matching chain lengths, their altered miscibility may be related to the 1-deoxy-Cer decreased amphipathicity and/or distorted H-bonding ability. Li *et al*. found that C-1 hydroxyl in Cer is involved in a network of cooperative H-bonds that involves both OH groups, NH group and two molecules of water^5^. Although that behavior was described in solution, it seems safe to assume that the lack of C-1 hydroxyl would strongly distort the Cer interfacial H-bond network in lipid membranes as well. A similar separation of 1-deoxy-Cer-rich aggregates was found in their mixtures with sphingomyelin^4^. We expect that the separated domains are distributed in each layer of the film and they are larger than a scale of a lamella. These domains should contain tens of repeating units as they were detected by XRD^57^. Notably, chain disorder is also an important aspect as it may facilitate the formation of larger domains and less defects. For example, our control samples with native Cer have 4–20% fluid and ~1% isotropic lipids at 32 °C as found by solid state NMR^58^.

Despite the extensive phase separation, the water loss through 1-deoxy-Cer lipid lamellae was similar to control. Using TH and IND as model permeants, 4–5-fold increase in permeability in 1-deoxy-Cer sample over control was found, which is a relatively mild effect compared to, *e.g*., 79-fold increased permeability of a lipid model with short chain Cer^13^. This relatively good barrier of the 1-deoxyCer model lipid film may be explained by the presence of well-ordered and tightly packed lipids in the individual domains as observed by infrared spectroscopy. In addition, the 1-deoxy-Cer-rich domains will also be extremely hydrophobic that would likely hamper water loss. Thus, the permeability markers may cross the SC lipids through the phase boundaries, which will likely be a less ordered but tortuous pathway. Nevertheless, the presence of 1-deoxy-Cer in the mixture likely reduced the number/size of defects compared to a similar system without Cer (Chol/LIG/CholS) that had 20-fold lower electrical impedance and ~10-fold higher permeability to TH^12^.

It should be noted that XRD and infrared experiments were measured at room humidity but the lipid films in the Franz cells were exposed to a hydration gradient. Thus, the permeation experiment mimics the physiological hydration which was not possible to reach in the other experiments. However, SC is a relatively dry tissue and water is located primarily in cells^59^. The SC lipids do not excessively hydrate and do not swell, contrary to phospholipids (2 water molecules bind per one lipid after 24 h equilibration at 100% relative humidity^60^). We also performed several control XRD measurements on similar lipid systems and found neither membrane swelling nor modification of repeat distance after hydration at ~100% relative humidity for 24 h^8,19^, or after several cycles of hydration at 100% relative humidity (unpublished results). In addition, full hydration also did not markedly change infrared spectra of SC lipid models^12^. However, Pham *et al*. reported that the water content in the SC changed the proportions of solid and fluid lipid chains in the SC^61^. Thus, studies investigating the hydration effects in SC or model membranes do not correlate and this point requires further attention.

### 3-deoxy-Cer

A nuclear magnetic resonance study showed that C-3 hydroxyl in (dihydro)sphingosine Cer participates in their H-bond network^5^. The C-3 hydroxyl group is also a required substituent for a sphingomyelinase substrate^62^. However, other studies suggested that the C-3 hydroxyl is not necessary for sphingolipid interactions with other lipids^6,63,64^. For example, 3-*O-*methylation of Cer did not markedly alter its ability to displace Chol from interactions with sphingomyelin^6^.

In our model SC lipid systems, 3-deoxy-Cer mixed with LIG and Chol and formed well-organized MLP. The formation of MLP was recently observed in lipid systems consisting of 6-hydroxysphingosine-Cer^9^, sphingosine-Cer^22^, and their mixture^10^. We assume that the principles of the organization of MLP are similar to *Lγ* phase with asymmetric bilayers described for phospholipids^65–68^ and to oriented fatty acid bilayers with well-defined asymmetric distribution of lipids^57^. MLP contains most likely an asymmetric unit arranged so that it provides the repeat distance over 10 nm^22^. However, the detail molecular arrangement of MLP is unknown. In contrast to 3-deoxy-Cer, a change of C-3 stereochemistry in Cer (from D-*erythro*-lignoceroyl sphingosine to its L-*threo*-isomer) did not affect the lamellar periodicity in the SC lipid models but diminished the Cer mixing with fatty acids and reduced the water permeability barrier of such lipid systems^23^. Thus, the removal of C-3 hydroxyl in Cer was apparently less detrimental for its mixing with fatty acids and the ability to create a water barrier than incorrect stereochemistry at this position.

The 4-fold increased permeability of the 3-deoxy-Cer-based systems relative to control is consistent with the barrier properties of another MLP-forming SC lipid model^9^. A conceivable explanation could be that MLP arrangement potentiates the permeation along the lipid lamellae. In contrast to the apparent resistance of both deoxy-Cer lipid assemblies to water loss, further hydroxylation of Cer, either in the sphingosine^9^ or acyl chain (Kováčik *et al*., unpublished data), increased water loss in similar model systems up to 2-fold compared to sphingosine Cer control.

### *N-*Me-Cer

The rigid planar amide group of Cer was found to be oriented perpendicular toward the axes of the two hydrocarbon chains^69^. This amide group appears to be fundamental for the sterol displacing ability of Cer in creating signal-transducing membrane platforms^6,70^. Cer *N*-methylation lowered its melting point by more than 40 °C, indicating strongly disturbed attractive forces between *N*-Me-Cer molecules compared to Cer. In the model SC lipid systems, Cer *N*-methylation resulted in less ordered and less tightly packed lipid chains and improved mixing with Chol (compared to Cer). Interestingly, although a significant portion of LIG molecules separated from the mixture, as indicated by FTIR results, some LIG was apparently required for dissolving Chol in the lipid system (as the binary mixture of *N*-Me-Cer/Chol did not fully mix). Whether the driving force for this behavior was the absence of hydrogen or the presence of more lipophilic and bulkier methyl at the amide nitrogen is unknown.

The lipids in this *N*-Me-Cer sample created SLP and a phase with a repeat period of 9.11 nm, which was likely rich in *N*-Me-Cer and Chol. SLP also contained some *N*-Me-Cer as Chol does not mix well with LIG. Raudenkolb *et al*. found a structure with a repeat distance of 7.4 nm in hydrated *N*-(α-hydroxyoctadecanoyl)-phytosphingosine with unnatural L-configuration in α-position. They proposed a repeat unit of two V-shaped Cer ordered alternatingly^28^. This principle of alternating V-shaped lipids could also be consistent with the 9.11 nm phase found here (note that our *N*-Me-Cer has by 6 C longer acyl chain, and the angle of the V-shape conformation could be wider resulting in a larger repeat distance compared to Raudenkolb *et al*.^28^).

Consequently, the lipid model with *N*-Me-Cer had approximately 10-fold higher permeabilities to TH and IND and 3-fold higher water loss compared to the model with physiological Cer. This barrier impairment, which is 2–3-fold greater compared to the effects of either deoxy-Cer, may be related to the less ordered, loosely packed lipids separated into two distinct phases (but not to macroscopic defects in the lipid film as indicated by the high electrical impedance value). A question remains how Chol fully mixed with other lipids affected the permeability of such lipid assemblies. We have previously described that a 0.4:1:1 molar ratio of Chol/Cer/fatty acids appears sufficient for skin lipids to limit water loss and prevent the entry of environmental substances and that excessive Chol phase separates and rather disturbs the barrier^55^.

### Conclusions

We have previously reported that the both acyl^12^ and sphingosine chains shortening^13^, saturation of *trans*-double bond^71^, hydroxylations in C-4^8,72^ or C-6 position^9,10^ have different effects on the phase behavior and permeabilities of model skin lipid mixtures. Here we focused on the essential molecular features common to all Cer subclasses, hydroxyls at C-1 and C-3 and amide bond. These Cer polar head group modifications had an immense impact on the studied properties of the SC model lipid mixtures. The hydroxyl group in C-1 position was necessary for proper lipid mixing as the resulting 1-deoxy-Cer-based systems were fragmented into several crystalline domains. This behavior most likely underlined the moderately worsened barrier function of 1-deoxy-Cer-based SC lipid barrier models compared to those with physiological Cer. In contrast, the 3-deoxy-Cer mixed well with other lipids and, at the conditions used here, formed MLP phase with 10.8 nm periodicity. Thus, the position of hydroxyl groups in the Cer polar head is decisive for their miscibility and lamellar phase arrangement. However, the lipid systems with 3-deoxy-Cer had similar permeabilities to those with 1-deoxy-Cer highlighting the complex relationships between the structure of a lipid assembly and permeability. Methylation of the Cer nitrogen improved Cer miscibility with Chol, disordered lipid chains, and strongly increased the permeability of the systems to all studied markers compared to the control with physiological Cer. In conclusion, hydroxyl in position 1 and monosubstituted amide bond in Cer should be maintained in rational designing of lipid analogs for barrier repair therapy of skin diseases, whereas the allylic hydroxyl in position 3 can possibly be removed/replaced.

## Materials and Methods

### Chemicals

1-Deoxy-sphingosine (synthetic, over 99% stereochemically pure, *i.e*., (2 *S*,3 *R*,4*E*)), 3-deoxy-sphingosine (synthetic, over 99% stereochemically pure, *i.e*., (2 *R*,4*E*)), *N*-methyl-sphingosine (synthetic, over 99% stereochemically pure, *i.e*., (2 *S*,3 *R*,4*E*)) and Cer (CerNS; synthetic, over 99% stereochemically pure, *i.e*., (2 *S*,3 *R*,4*E*)) were purchased from Avanti Polar Lipids (Alabaster, USA). Deuterated lignoceric acid (*d*-LIG) was obtained from C/D/N isotopes (Pointe-Claire, Canada). All other chemicals and solvents were from Sigma-Aldrich (Schnelldorf, Germany). Water was deionized, distilled, and filtered through a Millipore Q purification system.

### Synthesis of Cer analogs

See Supporting Information.

### Preparation of lipid models

The skin barrier models were prepared as equimolar mixtures of Cer or its unnatural analogs, Chol, and LIG with the addition of 5 wt% of CholS^9,46^. First, the lipids were dissolved in 2:1 hexane/96% EtOH (or 96% EtOH for CholS) with sonication and mixed to yield the desired composition. The lipid solutions were evaporated under a stream of nitrogen, dried under vacuum, and then redissolved in 2:1 hexane/96% ethanol (v/v) at 4.5 mg/mL. These lipid solutions (3 × 100 µL per cm^2^) were slowly sprayed on Nuclepore polycarbonate filters with 15 nm pores (Whatman, Kent, UK) or on 22 mm × 22 mm supporting glass cover slides under a stream of nitrogen using a Linomat V (Camag, Muttenz, Switzerland) equipped with additional y-axis movement. This fast drying suppressed artefactual lipid unmixing during solvent evaporation. The supporting filters did not significantly contribute to membrane barrier properties^8^. The lipid films were heated to 90 °C, a temperature that is above the main lipid phase transitions in our samples, equilibrated for 10 min, and then slowly (~3 h) cooled to room temperature. All samples were equilibrated at 32 °C for at least 3 days before the experiments.

### X-ray diffraction (XRD)

The XRD data on the lipid films on glass cover slides were collected at ambient room temperature and humidity with an X’Pert PRO θ-θ powder diffractometer (PANalytica B.V., Almelo, Netherlands) with parafocusing Bragg-Brentano geometry using CuKα radiation (λ = 1.5418 Å, U = 40 kV, I = 30 mA) in modified sample holders over the angular range of 0.6–30° (2θ). Data were scanned with an ultrafast linear (1D) position-sensitive X’Celerator detector with a step size of 0.0167° (2θ) and a counting time of 20.32 s step^−1^. The raw scattered intensities without normalization are shown as a function of the scattering vector Q [nm^−1^]. The data were evaluated using the software package X’Pert Data Viewer (PANalytical B.V., Almelo, Netherlands) as described previously^9,23^.

### Infrared spectroscopy

Fourier-transform infrared spectra of the model SC lipid mixtures were collected on a Nicolet FTIR 6700 spectrometer (Thermo Scientific, Waltham, MA, USA) using a single-reflection MIRacle attenuated total reflectance ZnSe crystal (PIKE technologies, Madison, WI, USA). A clamping mechanism with constant pressure was used. The spectra were generated by co-addition of 256 scans collected at 2 cm^−1^ resolution. The temperature dependence of infrared spectra was studied over the range 28–100 °C with 2 °C steps using a temperature control module (PIKE technologies, Madison, WI, USA). The data were analyzed using Bruker OPUS software.

### Permeation experiments

The permeability of the model SC lipid films was evaluated using Franz diffusion cells with an available diffusion area of 0.5 cm^2^ and an acceptor volume of 6.5 ± 0.1 mL. The acceptor compartment of the cell was filled with phosphate-buffered saline (PBS) at pH 7.4 with 50 mg/L gentamicin and stirred at 32 °C^8^. After a 24-h equilibration, we checked the samples for macroscopic defects using electrical impedance^11,73^. The electrical impedance was recorded using an LCR 4080 meter (Conrad Electronic, Hirschau, Germany) operated in parallel mode with an alternating frequency of 120 Hz. This setup yields the best sensitivity to small impedance changes. To record the membrane impedance, the donor compartment of the Franz diffusion cell was filled with 0.5 mL of PBS, and the tips of the stainless-steel probes were carefully immersed in PBS in the donor and acceptor compartments of the diffusion cell. Next, the membranes were equilibrated overnight and water loss [g/h/m^2^] through the lipid films was measured using Tewameter® TM 300 probe and Multi Probe Adapter Cutometer® MPA 580 (CK electronic GmbH, Kӧln, Germany)^23^. The environmental conditions were comparable during all measurements: ambient air temperature of 26–29 °C and relative air humidity of 40–46%. Next, 100 µL of the donor sample – either 5% TH or 2% IND suspensions in 60% propylene glycol – were applied to the lipid film. 60% propylene glycol does not extract lipids from the SC model membranes^19^ or change SC lipid chain order^74^. This setup ensured sink conditions for the selected drugs. Samples of the acceptor phase (300 µL) were withdrawn every 2 h over 8 h and were replaced with the same volume of PBS. During this period, a steady-state situation was reached. The acceptor phase samples were analyzed for TH and IND by HPLC using validated methods^8^.

### Data analysis

The cumulative amounts of TH and IND were calculated from the concentration measured by HPLC and diffusion cell volume, and were corrected for the acceptor phase replacement. The cumulative amounts were plotted against time and the steady-state flux of TH (or IND) was calculated as a slope of the linear regression function obtained by the fitting the linear region of the plot in Excel. Data are presented as the means ± SEM, and the number of replicates is given in the pertinent figure. One-way analysis of variance (ANOVA) with Dunnett’s post hoc test method was used for statistical analysis and *p* < 0.05 was considered significant.

## Supplementary information

Supplementary info.
