# Supplementary material for: Behavior of 1-Deoxy-, 3-Deoxy- and N-Methyl-Ceramides in Skin Barrier Lipid Models
Source: Sci Rep. 2020 Mar 2;10:3832. doi: 10.1038/s41598-020-60754-4 (PMC7051948; doi:10.1038/s41598-020-60754-4)
Supplement: Supplementary file 1 — Supplementary info. [file 41598_2020_60754_MOESM1_ESM.docx]

SUPPORTING INFORMATION

Behavior of 1-Deoxy-, 3-Deoxy- and N-Methyl-Ceramides
in Skin Barrier Lipid Models

Andrej Kováčik^†^, Petra Pullmannová^†^, Ludmila Pavlíková^†^, Jaroslav Maixner^§^, and Kateřina Vávrová^†^*

^†^Skin Barrier Research Group, Faculty of Pharmacy in Hradec Králové, Charles University, Akademika Heyrovského 1203, 50005 Hradec Králové, Czech Republic

^§^University of Chemistry and Technology in Prague, Faculty of Chemical Technology, Technická 5, 166 28 Prague, Czech Republic

* Corresponding author: [*katerina.vavrova@faf.cuni.cz*](mailto:katerina.vavrova@faf.cuni.cz)

**Table of contents:**

Synthesis of Cer Analogs

Figure S1 – Copies of NMR spectra

Figure S2 – Comparison of Cer melting points and transition temperatures of the lipid systems

Tables S1-S4 – Lists of XRD reflections

Figure S3 – XRD diffractograms of the unnatural Cer and their mixtures with Chol

Figure S4 – Temperature dependence of the infrared scissoring wavenumbers

Figure S5 – Infrared spectra of COOH and amide vibrations

**Synthesis of Cer Analogs**

Appropriate sphingoid base (0.053 mmol), lignoceric acid (LIG; 0.058 mmol) and 1-hydroxybenzotriazole hydrate (HOBt; 0.196 mmol) were dissolved in 5 mL of dry THF under argon and cooled to 0°C. Next, 1-ethyl-3-(3-dimethylaminopropyl)carbodiimide (WSC; 0.106 mmol) was slowly added and stirred for 0.5 h at 0°C. The reaction mixture was then allowed to warm to room temperature and stirred overnight. Next, the reaction mixture was evaporated; the residue was purified by column chromatography on silica gel (Merck Kieselgel 60, 0.040–0.063 mm) using 50:1 CHCl_3_/MeOH (v/v) as the mobile phase. For TLC, Merck TLC aluminum sheets with silica gel 60 F254 and ammonium molybdate with ceric sulfate in sulfuric acid were used. Infrared spectra were measured on a Nicolet 6700 in the ATR mode (Thermo Scientific, Waltham, MA, USA). ^1^H and ^13^C NMR spectra were recorded on a VNMR S500 NMR spectrometer (Palo Alto, CA, USA). Chemical shifts were reported as *δ* values in parts per million (ppm) and were indirectly referenced to tetramethylsilane (TMS) *via* the solvent signal. Mass spectrometry was measured on an LCQ Advantage Max (Thermo Finnigan, San Jose, USA) equipped with an APCI source.

*N*-((2*S*,3*R*,4*E*)-3-hydroxyoctadec-4-ene-2-yl)-tetracosanamide (**1-deoxy-Cer**). Yield = 94%, white crystals, R*f* = 0.80 (CHCl_3_/MeOH, 10:1), m.p. 92–94°C. ^1^H NMR (500 MHz, CDCl_3_/MeOD, 10:1): *δ* = 6.18 (d, *J* = 8.2 Hz, 1H), 5.70 – 5.62 (m, 1H), 5.36 (dd, *J* = 15.5, 6.5 Hz, 1H), 4.05 – 3.91 (m, 2H), 2.11 (t, *J* = 7.7 Hz, 2H), 2.02 – 1.95 (m, 2H), 1.60 – 1.48 (m, 2H), 1.20 (d, *J* = 1.8 Hz, 62H), 1.03 (d, *J* = 6.8 Hz, 3H), 0.83 (t, *J* = 6.9 Hz, 6H) ppm. ^13^C NMR (125 MHz, CDCl_3_/MeOD, 10:1): *δ* = 174.14, 133.75, 128.07, 74.91, 49.06, 36.57, 32.25, 31.78, 30.13, 29.90, 29.55, 29.51, 29.39, 29.37, 29.23, 29.21, 29.12, 29.09, 25.69, 22.53, 14.77 ppm. IR (ATR): *ν* = 3630, 2918, 2850, 1627, 1473, 1463, 730, 720 cm^-1^. MS (APCI^+^): *m/z* 634.8 (M+H^+^); 617.0 (M+H^+^ – H_2_O).

*N*-((2*R*,4*E*)-1-hydroxyoctadecan-4-ene-2-yl)-tetracosanamide (**3-deoxy-Cer**). Yield = 69%, white crystals, R*f* = 0.75 (CHCl_3_/MeOH, 10:1), m.p. 81–84°C. ^1^H NMR (500 MHz, CDCl_3_/MeOD, 10:1): *δ* = 6.06 (d, *J* = 7.8 Hz, 1H), 5.54 – 5.41 (m, 1H), 5.36 – 5.23 (m, 1H), 3.89 – 3.78 (m, 1H), 3.55 (dd, *J* = 11.2, 4.3 Hz, 1H), 3.48 (dd, *J* = 11.2, 5.8 Hz, 1H), 2.28 – 2.17 (m, 1H), 2.13 (t, *J* = 7.6 Hz, 2H), 2.02 – 1.90 (m, 1H), 1.62 – 1.50 (m, 2H), 1.34 – 1.12 (m, 64H), 0.84 (t, *J* = 6.9 Hz, 6H) ppm. ^13^C NMR (125 MHz, CDCl_3_/MeOD, 10:1): *δ* = 174.42, 134.42, 125.00, 64.39, 51.17, 36.65, 34.13, 32.49, 31.81, 29.59, 29.54, 29.43, 29.41, 29.37, 29.27, 29.24, 29.15, 29.13, 25.73, 22.57, 13.96 ppm. IR (ATR): *ν* = 3360, 2918, 2850, 1655, 1560, 1469, 720 cm^-1^. MS (APCI^+^): *m/z* 634.9 (M+H^+^); 616.9 (M+H^+^– H_2_O).

*N*-((2*R*,3*R,*4*E*)-1,3-dihydroxyoctadecan-4-ene-2-yl)-*N*-methyltetracosanamide (***N*-Me-Cer**). Yield = 71%, white crystals, R*f* = 0.25 (CHCl_3_/MeOH, 10:1), m.p. 46–48°C. ^1^H NMR (500 MHz, CDCl_3_/MeOD, 10:1): *δ* = 5.73 – 5.58 (m, 1H), 5.39 (dd, *J* = 15.3, 8.0 Hz, 1H), 4.29 – 4.22 (m, 1H), 4.06 – 3.96 (m, 1H), 3.87 (dd, *J* = 12.0, 4.2 Hz, 1H), 3.81 (dd, *J* = 12.0, 7.3 Hz, 1H), 2.91 (s, 3H), 2.24 (t, *J* = 7.7 Hz, 2H), 2.02 – 1.91 (m, 2H), 1.61 – 1.46 (m, 2H), 1.38 – 1.07 (m, 62H), 0.84 (t, *J* = 6.9 Hz, 6H) ppm. ^13^C NMR (125 MHz, CDCl_3_/MeOD, 10:1): *δ* = 175.44, 134.24, 129.52, 72.13, 71.82, 62.57, 60.94, 34.19, 31.81, 29.60, 29.58, 29.55, 29.53, 29.50, 29.46, 29.43, 29.37, 29.25, 29.17, 29.13, 25.32, 24.94, 22.57, 13.96 ppm. IR (ATR): *ν* = 3357, 2918, 2850, 1617, 1467, 721 cm^-1^. MS (APCI^+^): *m/z* 647.0 (M+H^+^ – H_2_O); 628.9 (M+H^+^ – 2H_2_O).

**Figure S1.** NMR spectra of the synthesized 1-deoxy-Cer, 3-deoxy-Cer, and *N*-Me-Cer.


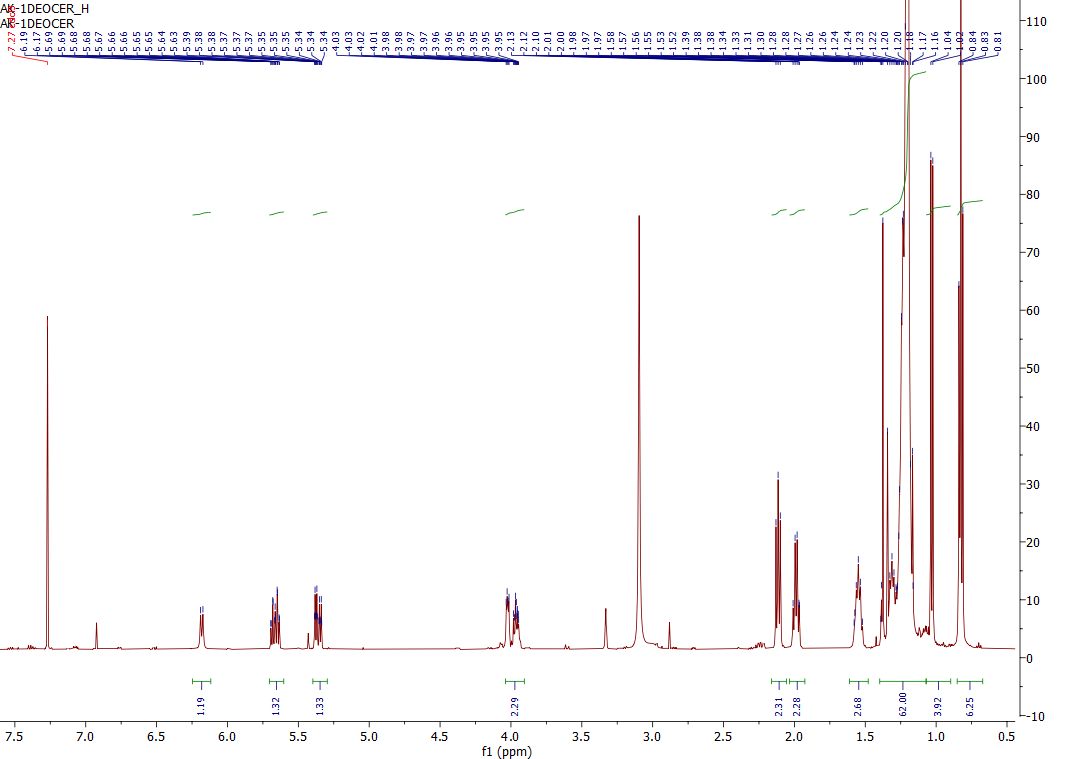


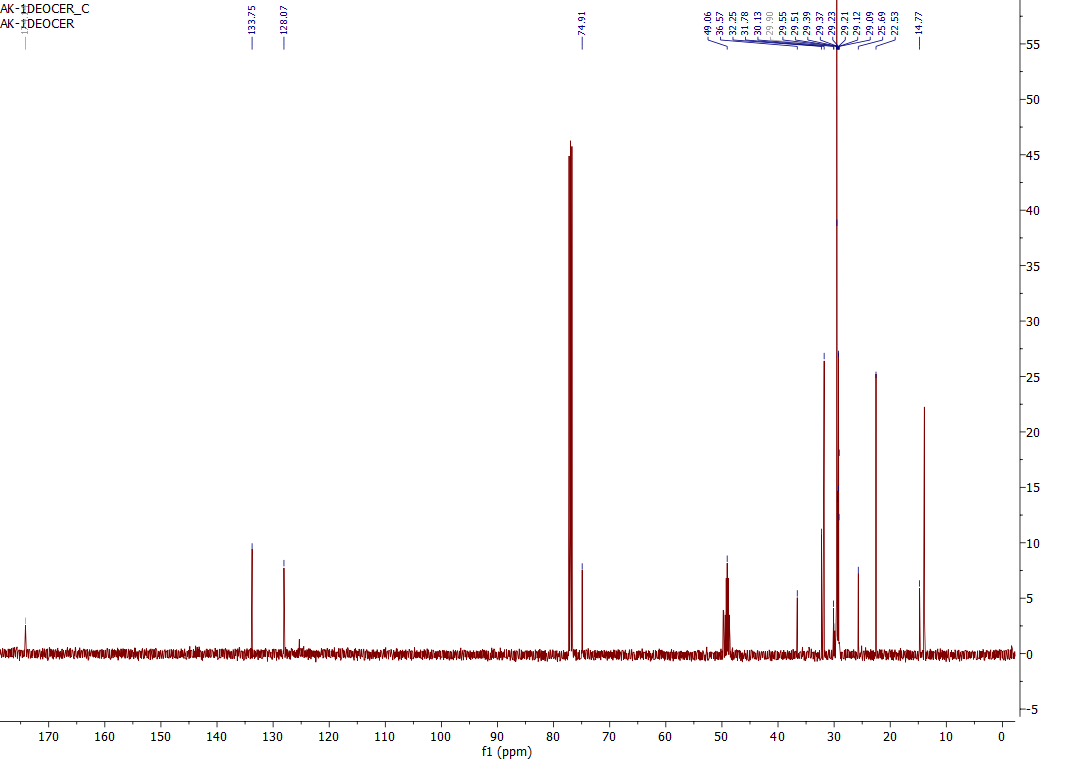


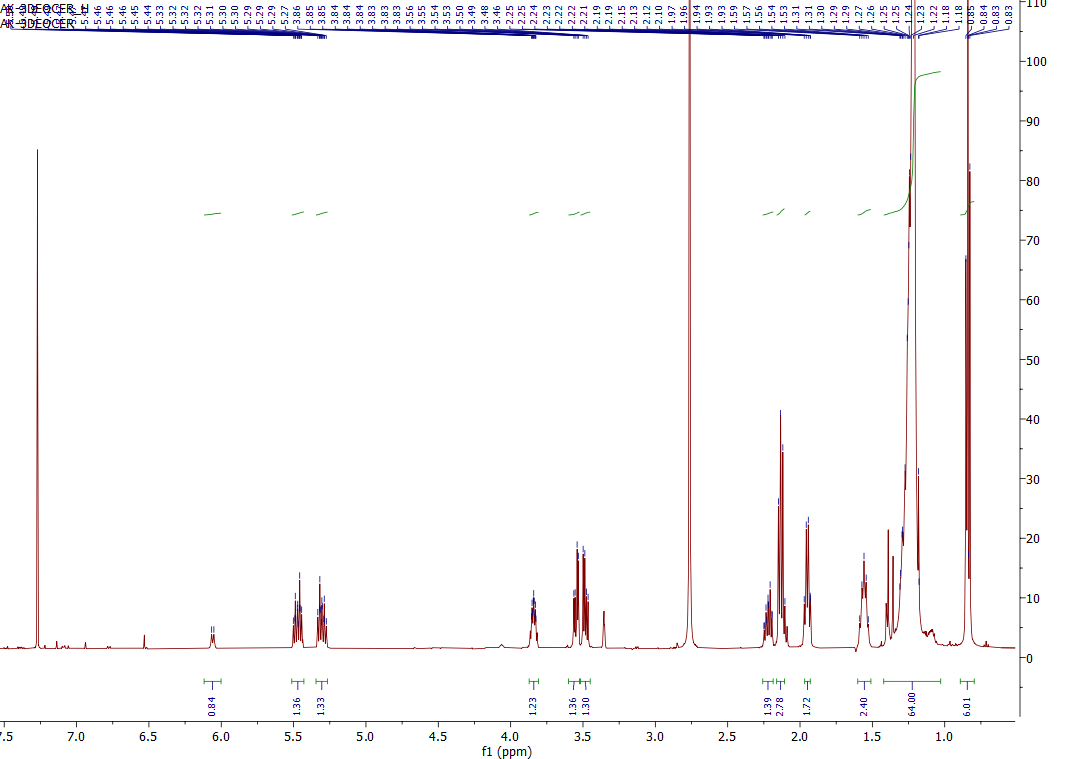


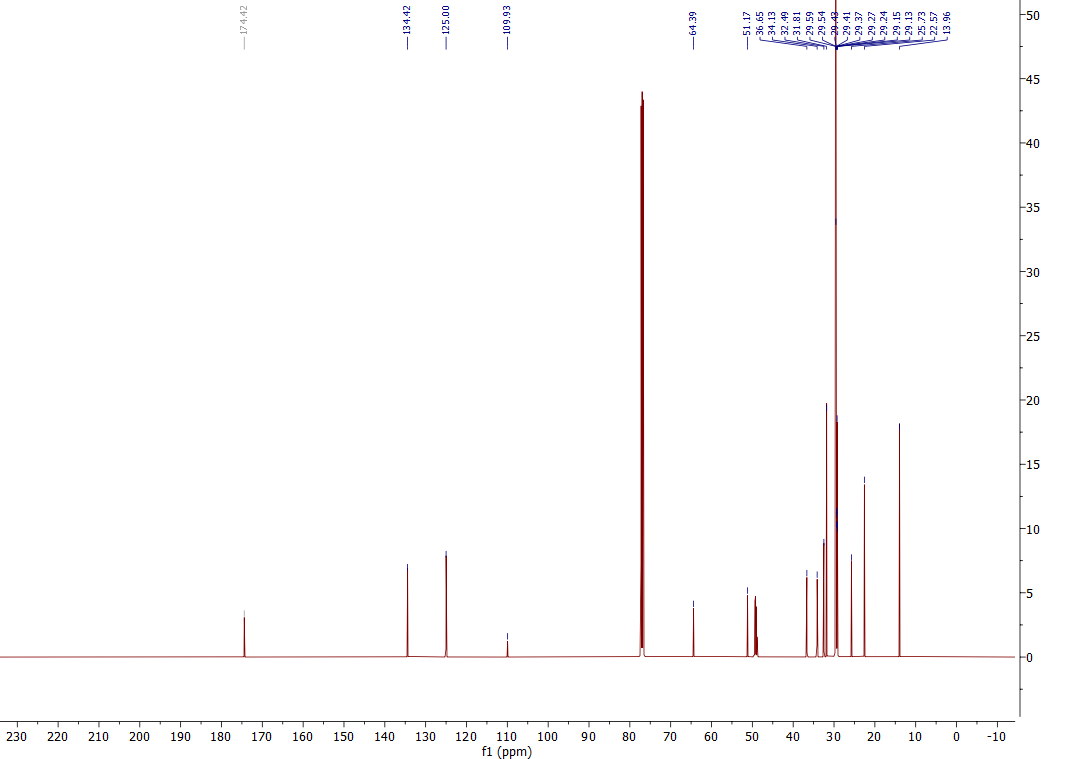


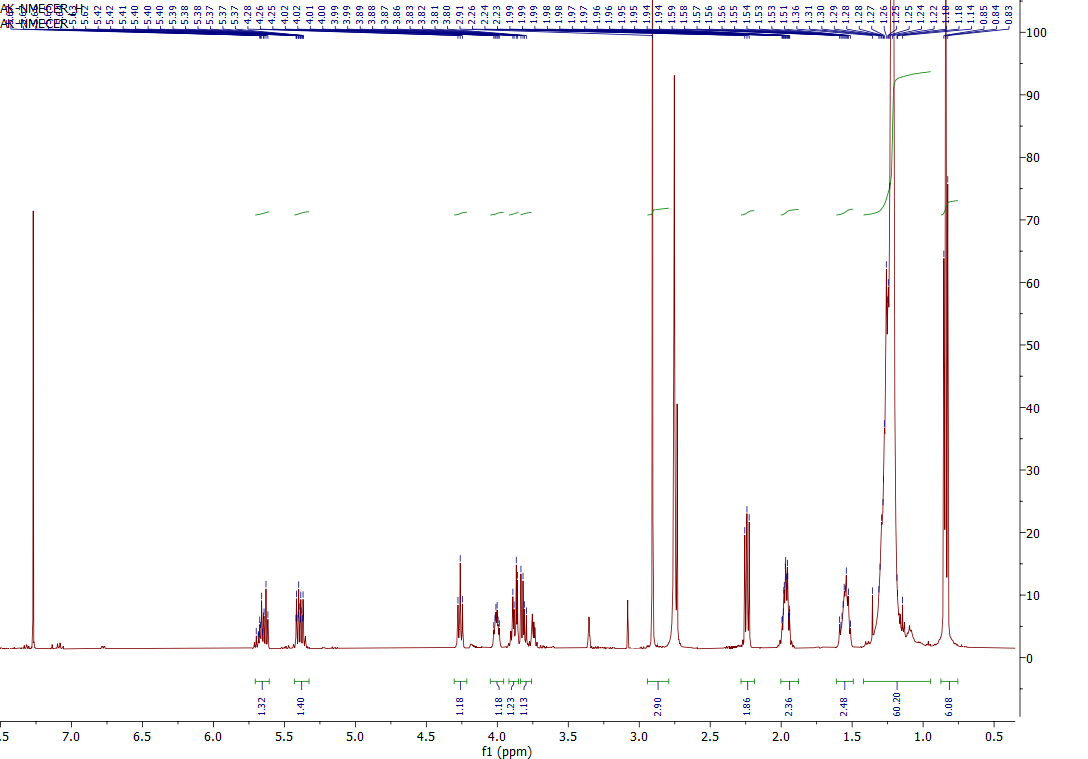


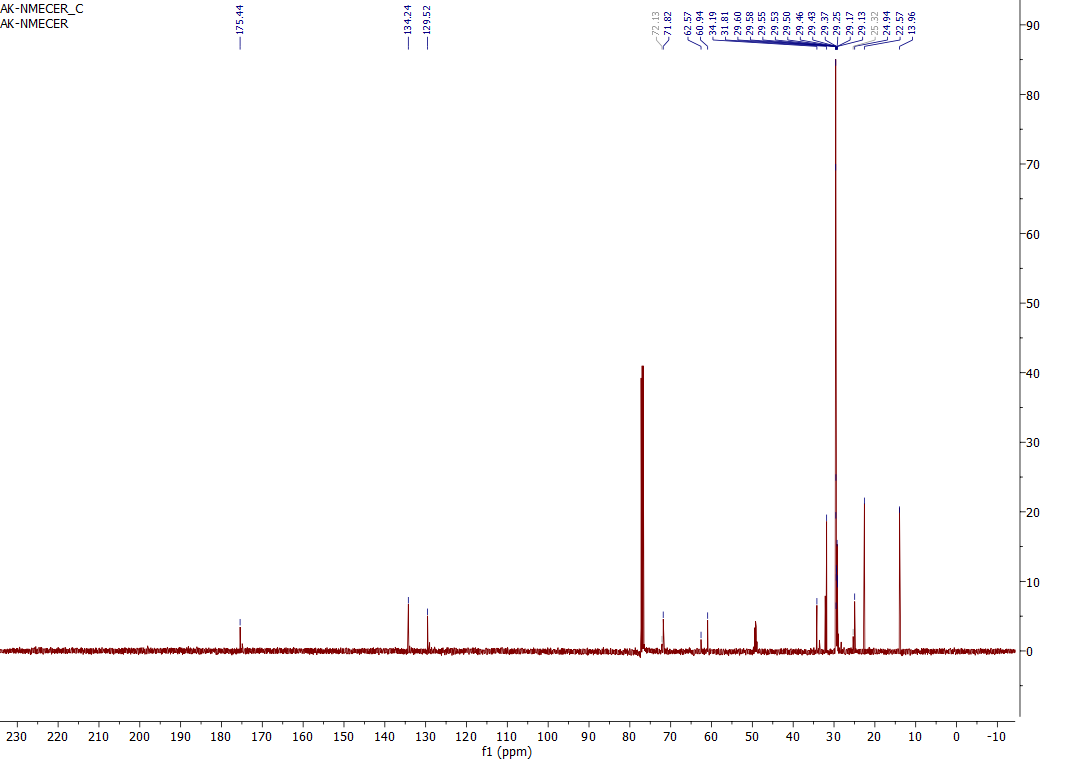


**
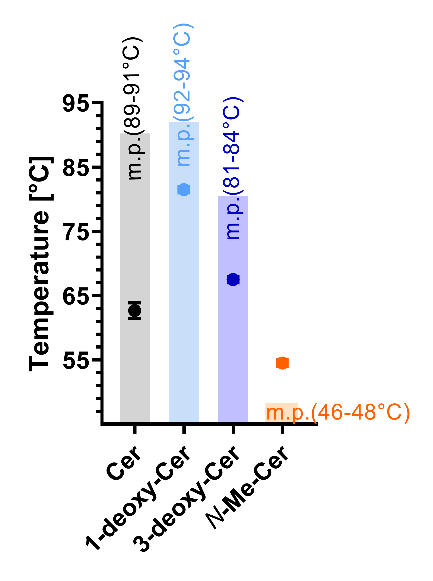
**

**Figure S2**. The comparison of the main phase transition temperatures of CH_2_ symmetric stretching vibrations (circles, mostly Cer chains) in samples with *d*-LIG and melting temperatures of pure Cer and unnatural Cer analogues (bars).

**Table S1.** List of XRD reflections in control Cer samples.

| **Sample** | **Lamellar phases** | | | **Chol** | **Region of short-range arrangement** |
| --- | --- | --- | --- | --- | --- |
|  | ***d* [nm]** | **Q [nm^-1^]** | **Q [nm^-1^]** | | **Q [nm^-1^]** |
| Cer/LIG/Chol/CholS  (sample 1) | 5.41 | 1.16, 2.33, 3.50, 4.66, 6.98, 8.14 | 1.84, 3.68 | | 15.15, 16.77 |
| Cer/LIG/Chol/CholS  (sample 2) | 5.40 | 1.17, 2.33, 3.49, 4.66, 6.98, 8.15, 9.30, 10.47 | 1.84, 3.68, 11.03 | | 15.17, 16.79 |

**Table S2.** List of XRD reflections in 1-deoxy-Cer samples.

| **Sample** | **Lamellar phases** | | | **Chol** | **Region of short-range arrangement** |
| --- | --- | --- | --- | --- | --- |
|  | ***d* [nm]** | **Q [nm^-1^]** | **Q [nm^-1^]** | | **Q [nm^-1^]** |
| 1-deoxy-Cer pure (sample 1) | 4.72 | 1.37, 2.69, 4.02, 5.35, 8.01, 9.34 | - | | - |
| 1-deoxy-Cer pure  (sample 2) | 4.73 | 1.37, 2.69, 4.02, 5.34, 8.01, 9.34 | - | | - |
| 1-deoxy-Cer/Chol  (sample 1) | 4.73 | 1.36, 2.68, 4.01, 5.34, 8.00 | 1.86, 3.69 | | - |
|  | - | **4.60** |  |  |  |
| 1-deoxy-Cer/Chol  (sample 2) | 4.73 | 1.34, 2.66, 3.99, 5.32, 7.98 | 1.84, 3.67 | | - |
|  | - | **4.58** |  |  |  |
| 1-deoxy-Cer/ LIG/Chol/CholS  (sample 1) | 4.71 | 1.39, 2.71, 4.04, 5.37, 6.77, 8.04 | 1.90, 3.73, 7.61, 9.23, 10.33, 11.07, 11.27, 12.10 | | 15.32, 16.51, 17.24, 20.20 |
|  | 5.26 | 1.27, 2.45, 3.65, 4.83, 6.04, 8.43, 9.62 |  |  |  |
|  | - | **1.53, 4.64** |  |  |  |
| 1-deoxy-Cer/ LIG/Chol/CholS  (sample 2) | 4.68 | 1.32, 2.65, 3.98, 5.31, 6.72, 12.05 | 1.84, 3.67, 5.54, 7.56  7.97, 9.16, 9.57, 9.76, 9.89, 10.26, 11.01, 11.22 | | 15.27, 16.45, 17.18 |
|  | 5.25 | 1.20, 2.40, 3.59, 4.76, 5.98, 8.39 |  |  |  |
|  | - | **1.45, 3.02, 4.59** |  |  |  |

**Table S3.** List of XRD reflections in 3-deoxy-Cer samples.

| **Sample** | **Lamellar phases** | | | **Chol** | **Region of short-range arrangement** |
| --- | --- | --- | --- | --- | --- |
|  | ***d* [nm]** | **Q [nm^-1^]** | **Q [nm^-1^]** | | **Q [nm^-1^]** |
| 3-deoxy-Cer pure (sample 1) | 5.55 | 1.15, 2.28, 3.40, 4.54, 6.76, 7.94, 10.13, 11.34, 13.60, 15.90 | - | | 14.98, 15.90, 16.17, 17.16, 17.46 |
|  | 3.72 | 1.70, 5.07, 8.45 |  |  |  |
| 3-deoxy-Cer pure  (sample 2) | 5.54 | 1.15, 2.28, 3.40, 4.54, 6.76, 7.94, 10.13, 11.34, 13.61, 15.91 | - | | 14.99, 15.91, 16.18, 17.17, 17.48 |
|  | 3.73 | 1.70, 5.07, 8.44 |  |  |  |
| 3-deoxy-Cer/Chol  (sample 1) | 5.58 | 1.16, 3.40, 6.78 | 1.87, 3.70, 11.03 | | - |
|  | 3.72 | 1.72, 5.10 |  |  |  |
| 3-deoxy-Cer/Chol  (sample 2) | 5.60 | 1.16, 3.40, 6.78 | 1.86, 3.69, 9.19, 11.03 | | - |
|  | 3.72 | 1.71, 5.09 |  |  |  |
| 3-deoxy-Cer/ LIG/Chol/CholS  (sample 1) | 10.80 | 1.22, 1.80, 2.38, 2.96, 3.54, 4.12, 4.71, 5.28, 5.87, 6.45, 7.03, 8.20, 9.36, 10.53 | 1.88, 3.71, 6.76, 11.04 | | 15.20, 16.82 |
|  | - | **1.48** |  |  |  |
| 3-deoxy-Cer/ LIG/Chol/CholS  (sample 2) | 10.80 | 1.18, 2.33, 2.91, 3.49, 4.07, 4.66, 5.24, 5.83, 6.98, 8.14, 9.30, 10.48 | 1.83, 3.65, 6.71, 10.99 | | 15.15, 16.77, 20.17 |
|  | - | **1.45** |  |  |  |

**Table S4.** List of XRD reflections in *N-*Me-Cer samples.

| **Sample** | **Lamellar phases** | | | **Chol** | **Region of short-range arrangement** |
| --- | --- | --- | --- | --- | --- |
|  | ***d* [nm]** | **Q [nm^-1^]** | **Q [nm^-1^]** | | **Q [nm^-1^]** |
| *N*-Me-Cer pure (sample 1) | 5.45 | 1.18, 2.33, 3.47, 4.62, 5.77, 8.09 | - | | - |
| *N*-Me-Cer pure  (sample 2) | 5.47 | 1.17, 2.32, 3.46, 4.62, 5.76, 8.07 |  | |  |
| *N*-Me-Cer /Chol  (sample 1) | 5.43 | 1.20, 2.35, 3.51, 4.67, 5.81, 8.14, 9.29 | 1.88, 3.73, 11.03, 11.14 | | 14.85, 16.70 |
|  | 9.20 | 1.35, 2.08, 2.73, 4.07, 4.79, 5.44, 6.17, 7.51 |  |  |  |
|  | - | **3.04** |  |  |  |
| *N*-Me-Cer /Chol  (sample 2) | 5.43 | 1.20, 2.35, 3.51, 4.67, 5.81, 8.14, 9.29 | 1.88, 3.73, 11.03, 11.14 | | 14.85, 16.69 |
|  | 9.20 | 1.35, 2.08, 2.73, 4.07, 4.79, 5.44, 6.17, 7.51 |  |  |  |
|  | - | **3.04** |  |  |  |
| *N*-Me-Cer/ LIG/Chol/CholS  (sample 1) | 5.35 | 1.22, 2.39, 3.56, 4.74 |  | | 15.10, 16.69 |
|  | 9.12 | 1.44, 2.11, 2.84, 4.23, 4.86, 5.60, 6.25, 7.64 |  |  |  |
|  | - | **2.92, 3.10, 6.76, 7.05** |  |  |  |
| *N*-Me-Cer/ LIG/Chol/CholS  (sample 2) | 5.35 | 1.18, 2.35, 3.52, 4.70 |  | | 15.07, 16.53 |
|  | 8.98 | 1.39, 2.06, 2.79, 4.19, 4.82, 5.58, 7.00, 7.59 |  |  |  |
|  | - | **1.33, 2.89, 3.06, 6.71, 7.07** |  |  |  |


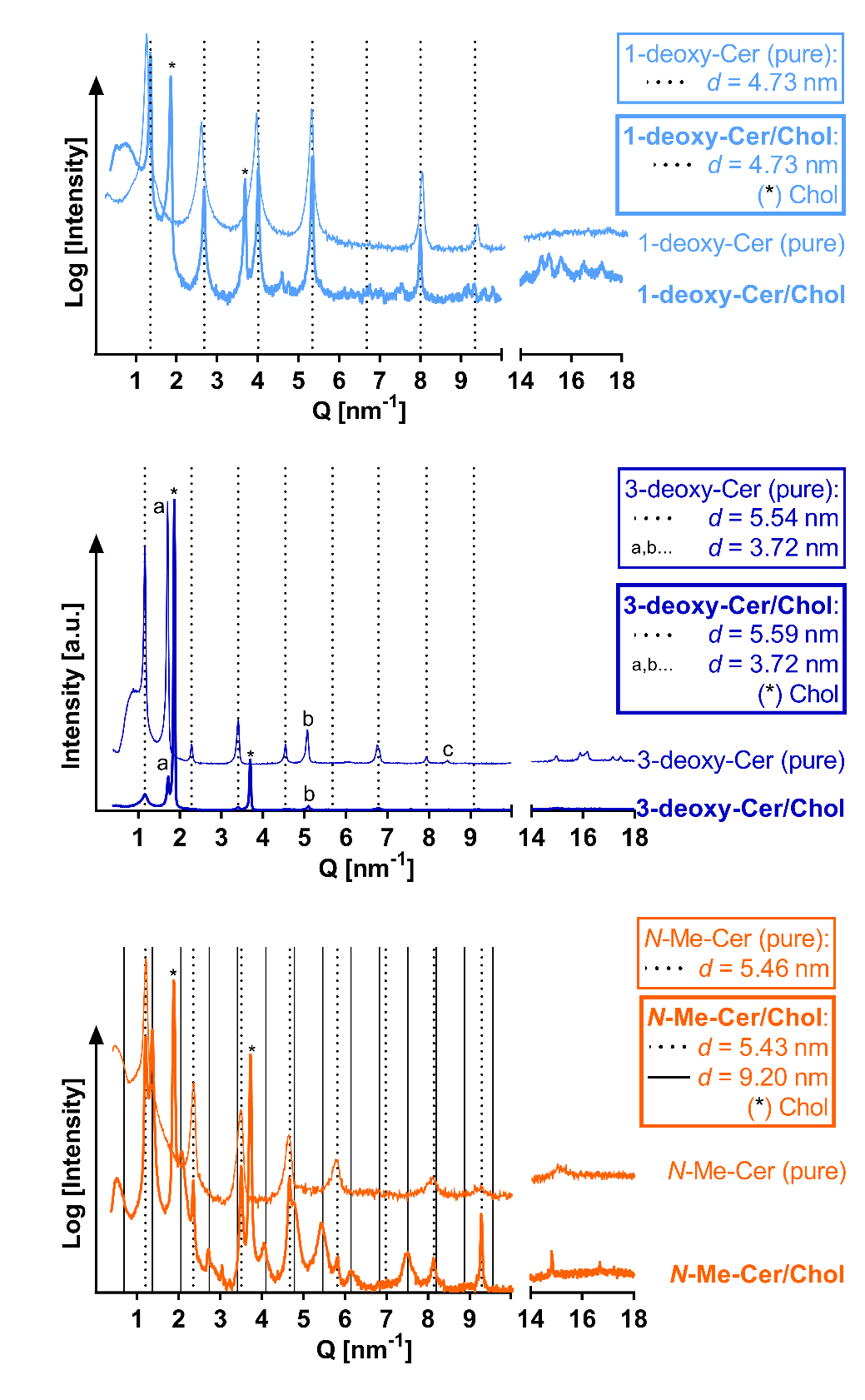


**Figure S3**. XRD diffractograms of the SC lipid models containing either pure unnatural Cer (light blue, 1-deoxy-Cer; dark blue, 3-deoxy-Cer; orange, *N*-Me-Cer) or a molar mixture of unnatural Cer and Chol (1:1). The intensity is given in arbitrary units (a. u.) and it is in logarithmic scale for 1-deoxy-Cer and *N*-Me-Cer. Full and dashed grind lines predict the positions of reflections providing the specified repeat distances. Letters mark additional reflections giving *d* = 3.72 nm in the 3-deoxy-Cer lipid mixture; asterisks mark the separated Chol.


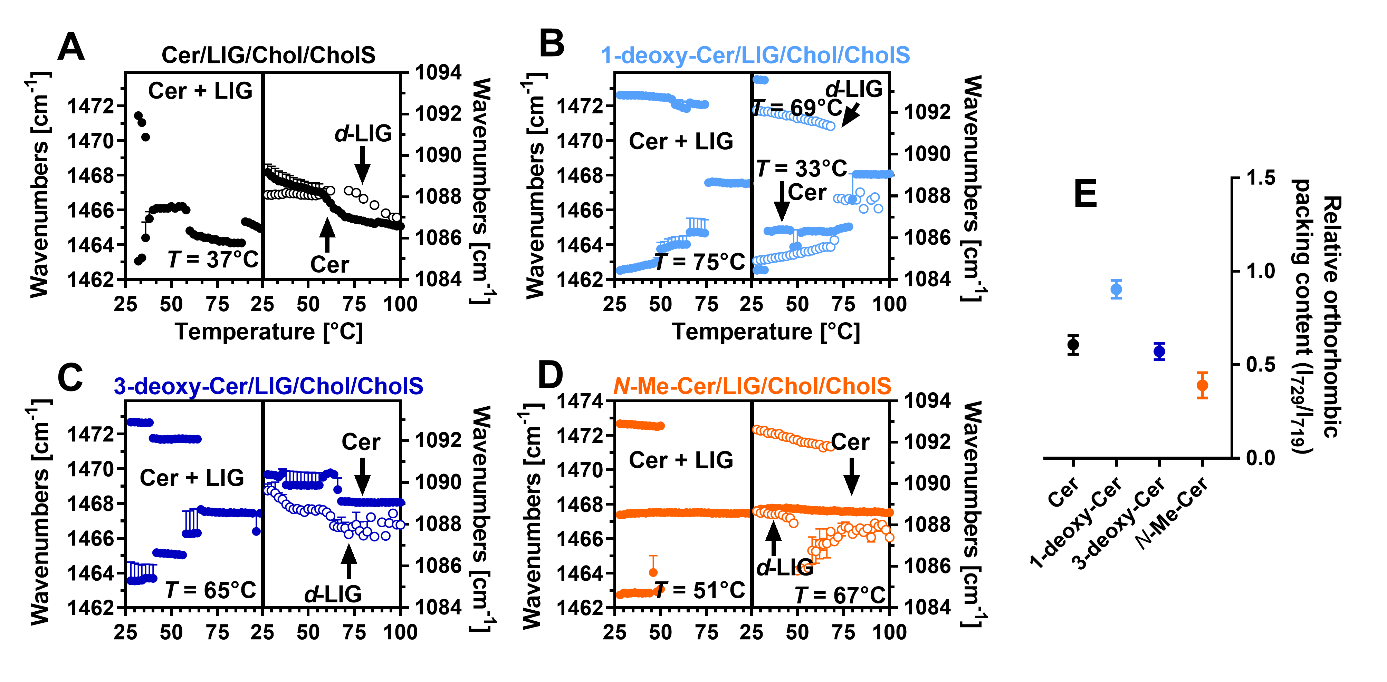


**Figure S4**. Lateral lipid packing in the SC lipid models containing the studied either Cer (black in panel A), or 1-deoxy-Cer (blue in panel B), or 3-deoxy-Cer (dark blue in panel C), or *N*-Me-Cer (orange in panel D), (*d*)-LIG, Chol (1:1:1 mol), and CholS (5 wt%). Unlabeled lipid mixtures: first graphs in each panel show the infrared spectra of the thermal evolution (25–100°C) of methylene scissoring vibrations. Lipid mixtures with *d*-LIG: second graphs show the CH*_2_* scissoring vibrations (mainly from Cers; filled circles) and the CD_2_ scissoring vibrations (from *d*-LIG; open circles). Two circles at a given temperature represent splitting of the scissoring band into a doublet, which is indicative of an orthorhombic structure. The temperatures of the apparent phase transitions are indicated. Panel E shows the relative orthorhombic packing ratios estimated from the rocking band (ratios of the 729–719 cm^−1^ peak intensities).


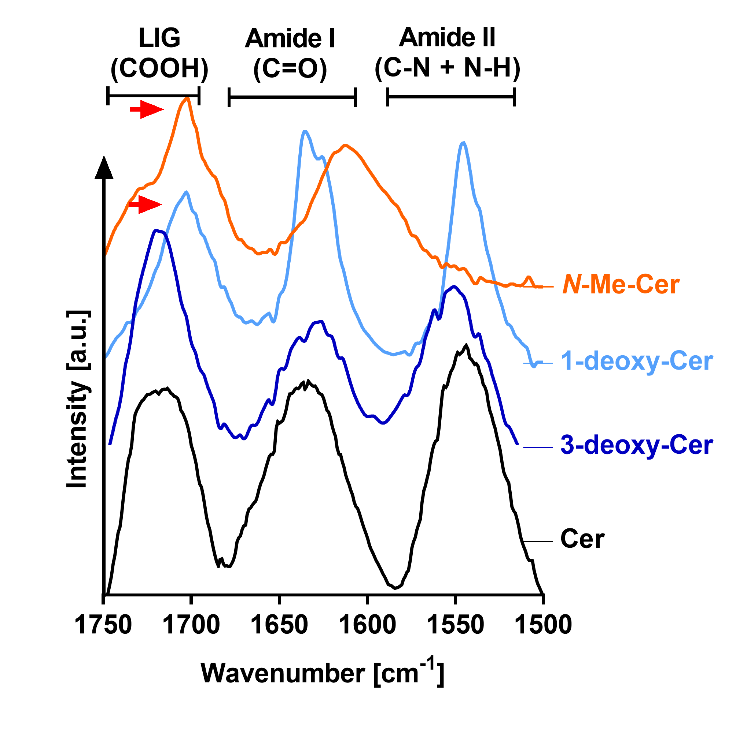


**Figure S5**. Infrared spectra of the polar head region of the SC lipid models composed of Cer (black), or 1-deoxy-Cer (light blue), or 3-deoxy-Cer (dark blue), or *N*-Me-Cer (orange), along with *d*-LIG, Chol, and CholS. Acid carbonyl stretching mode (~1700 cm^-1^), amide I (~1640 cm^-1^), amide II (~1550 cm^-1^) band vibrations at physiological temperature of 32°C are shown. Intensity is given in arbitrary units (a. u.).
